# A clinical trial evaluation of handwashing products and educational resources to improve hand hygiene in paediatric patients and school children

**DOI:** 10.3389/fpubh.2024.1427749

**Published:** 2024-09-23

**Authors:** Johanna McNicholl, Sarah Younie, Sapphire Crosby, Katie Laird

**Affiliations:** ^1^Faculty of Health and Life Sciences, Leicester School of Pharmacy, De Montfort University, The Gateway, Leicester, United Kingdom; ^2^Faculty of Health and Life Sciences, School of Applied Social Sciences, De Montfort University, The Gateway, Leicester, United Kingdom

**Keywords:** hand hygiene, paediatric patients, school pupils, educational resources, clinical trials, hand hygiene products

## Abstract

**Introduction:**

It is widely acknowledged that good hand hygiene (HH) is an important non-pharmaceutical method for reducing the transmission of infectious diseases. Children are at high risk of infection due to their immature immune systems. Hospital transmitted infections are a cause for concern worldwide, with poor HH suggested to be responsible for up to 20% of cases. Patients, in particular paediatric patients, are often overlooked when it comes to the promotion of hand hygiene compliance (HHC) in hospitals. This report describes the clinical evaluation of the ‘Soaper Stars’; a collection of child-friendly HH products with linked educational resource, developed using the COM-B approach to behaviour change, and designed to encourage correct HH in paediatric patients and in schools.

**Method:**

The Soaper Star products were distributed on paediatric wards in five UK hospitals, and the use of the products around mealtimes was evaluated. Workshops teaching the ‘why when and how’ of handwashing were run in four UK primary schools with pre and post evaluations conducted to establish impact on knowledge. Over 300 children were involved.

**Results:**

The Soaper Stars products stimulated a 38% increase in HHC compared to when only hospital-issued products were available, and verbal feedback from families indicated that having the Soaper Star products encouraged improved HHC by all visitors, not just the patient. Workshops in four schools (283 pupils) showed an increase in knowledge around the transmission of infection and the need for good HH that was sustained for at least 4 weeks.

**Conclusion:**

The results of this study demonstrate that providing children with the age-appropriate knowledge about why HH is necessary, and the child-friendly means to maintain their HH, will lead to greater HHC, not just by individual children, but also their families.

## 1 Introduction

In their 2022 report on global infection prevention (IP), the World Health Organisation (WHO) estimated that 7–15% of acute-care patients will acquire a healthcare-associated infection (HAI), rising to 30% for intensive care patients. The number of cases in lower-and middle-income countries (LMIC) may be 20 times that of a higher income country (HIC), especially in neonates (1). Children are particularly at risk of infectious diseases due to their underdeveloped immune systems (2), with recent mortality figures from UNICEF stating that globally, over 5 million children under five-years died in 2021, primarily due to infectious diseases (3). The WHO recommends hand hygiene as “*the most effective single measure to reduce the transmission of microorganisms/pathogens and infection in health care settings*” (1). However, there are limited studies or educational interventions that specifically target young children’s awareness of pathogen transmission, and hand-hygiene practices (4).

A lack of hand-hygiene compliance (HHC) within hospitals is said to be linked with the transmission of HAIs with HAI transmission predominately being via healthcare workers (HCW) (5). It is estimated that, annually, there are 300,000 HAIs, yet, with the correct infection control practices, 20% of these infections could be prevented (6). Studies regarding the transmission (and, prevention of transmission) of HAIs, has previously focused on the hand hygiene (HH) practices of HCW with mixed success (7).

Hospital patients have been described by Banfield and Kerr (8) as an ‘overlooked but potentially significant link’ in the chain of hospital acquired infection transmission. Historically, there has been limited published research with regards to the impact of patient hand-hygiene practices. More emphasis is now being placed on educating visitors and patients about the importance of correct handwashing practices (9–14).

Strategies to improve the HH of visitors to hospitals have included posters (11), verbal reminders and stickers (9), linking entry to wards with the use of alcohol hand sanitizer (13), moving hand sanitiser dispensers around different entry points of the hospital (15), education or verbal instructions from ward staff (10, 14) and using Glow-gel to teach handwashing techniques (12). Such interventions initially result in an improvement in HHC, few of the interventions have resulted in maintained improvements in HH for extended time periods, and consequently the increase in handwashing is not sustained.

In many countries, a significant proportion of patient care is provided by family members (16, 17). It is being recognised that family carers, other visitors to hospitals, and the patients themselves have a role to play in the transmission of HAIs (18).

A pre-COVID survey conducted to measure the attitudes of patients in relation to HH within hospitals found that patients were missing opportunities to complete HH practices, yet did not want to receive guidance to help them improve this. Some of the patients surveyed thought that they did not need to wash their hands while in a hospital bed, demonstrating a lack of awareness with regards to the transmission of pathogens (19).

While hand-hygiene education is important to limit the transmission of infections in a healthcare environment, children also need to understand the importance outside of a healthcare environment. To ensure that this happens, HH learning must be formally embedded from an early age (20, 21).

The UK National Curriculum for schools highlights the importance of HH learning for children. Teachers are required to deliver HH as part of the health education programme. The guidance states: ‘By the end of primary school, pupils should know about personal hygiene and germs including bacteria, viruses, how they are spread and treated, and the importance of hand washing’ (22).

Previous studies have shown the effectiveness of HH educational interventions within schools, in reducing the transmission of infection and maintaining attendance levels (23), with various strategies being used such as lesson plans (23, 24), child-designed posters in toilets and public spaces (25), promoting the use of alcohol-based hand sanitiser to reduce respiratory infections (26), carousel-style educational workshops (4, 27–29) handwashing songs and videos (30), and therapeutic clowning (31).

In addition to having suitable facilities for handwashing, such as water, soap, and basins, the attitudes and behaviour of other children is a significant influence on children’s motivation to complete HH. In Germany, a pre-COVID survey of 200 pupils aged 8–11 years found that almost 80% of them did not use toilet facilities at their school because of unsanitary conditions and/or the behaviour of their peers (32). Similarly, 79% of students surveyed in Faridabad, Northern India said they were more likely to wash their hands if they saw their friends also doing so (33). For 42% of the 670 pupils surveyed in Harar, eastern Ethiopia, the most common reason for not washing their hands was that they forgot to (34).

By delivering health-education interventions, it is thought children will be more likely to wash their hands more frequently and more effectively, if they are not only told how to do this, but also why they should do this.

This paper describes the creation and evaluation of the Soaper Stars, a child-focused educational intervention that can be used in hospitals and schools to promote good HH practices in children. Germ’s Journey co-creates, with end-users and specialists (scientists, educationalists, healthcare professionals, psychologists, and graphic designers) global educational resources that teach children about IP, hand-hygiene and pathogen transmission. After establishing a working relationship with PAL International (hygiene and infection control products manufacturers), the two teams collaborated to design the Soaper Star boxes.

The Soaper Stars are a collection of children’s characters, designed by the Germ’s Journey team, linked to HH products that have been designed to make engagement with HH fun and interactive for children, particularly those aged 6–10 years of age.

**Figure 1:**
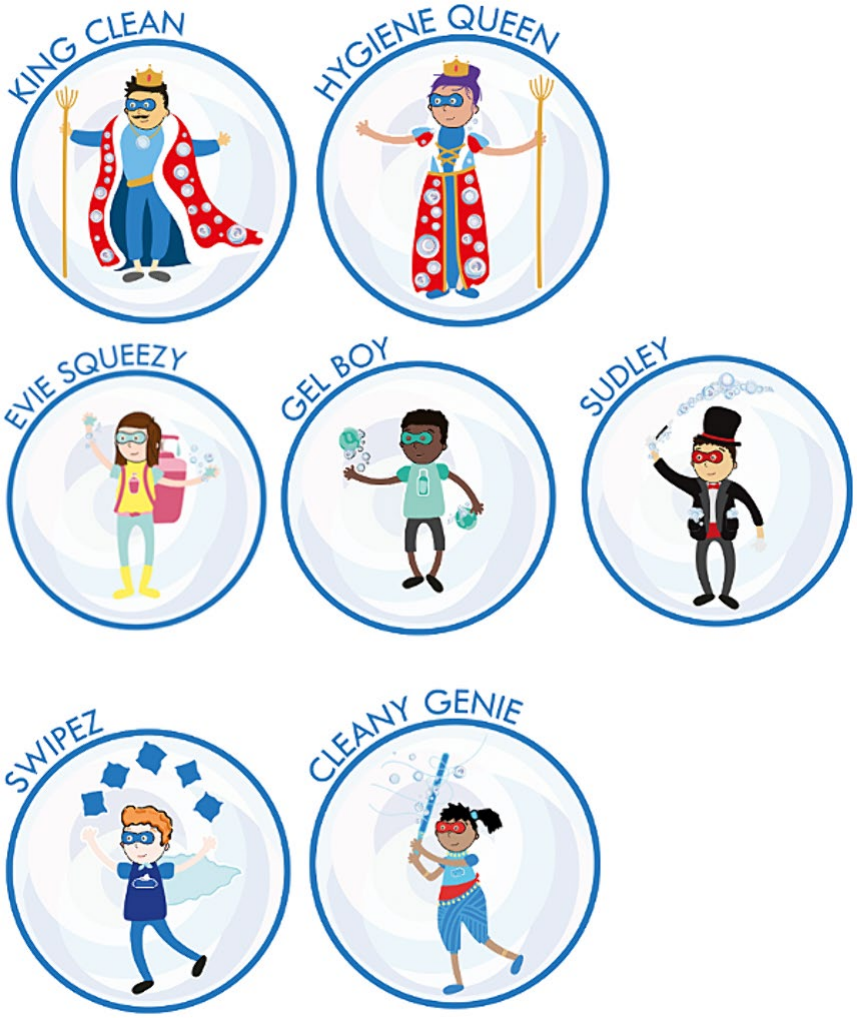
Soaper Stars characters.

Small boxes containing products (liquid soap, hand sanitiser and wipes) and educational resources (activity sheet containing activities and puzzles about the why, when and how of handwashing, a handwashing poster, colouring pencils and a sticker) were developed incorporating the Soaper Star characters (Figure 1). The boxes are ideally sized for storage in hospital bedside lockers and the products will fit into school bags or wash kits. The Soaper Stars were designed to be used in hospitals and schools to encourage children to partake in good HH, even in situations where access to handwashing facilities may be difficult.

## 2 Research questions

- Do the Soaper Star HH Boxes increase the frequency of paediatric patients’ hand-hygiene practice within hospitals?
- Do the Soaper Star HH Boxes give paediatric patients a greater awareness of hand-hygiene and IP?
- Do the Soaper Stars school workshops improve pupils’ understanding of hand-hygiene and IP?

## 3 Materials and methods

**Figure 2:**
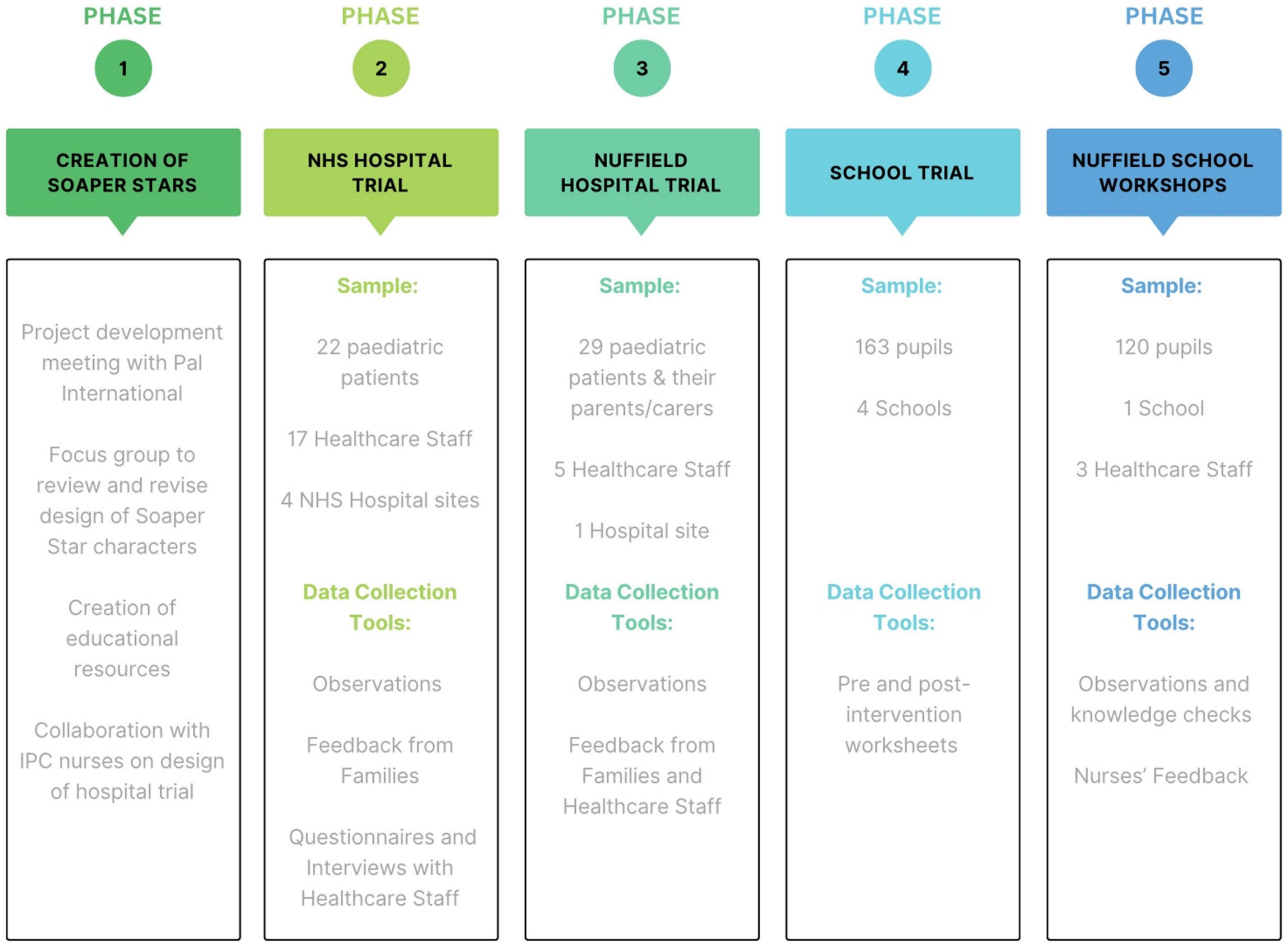
Project Overview: The project is divided into five phases. Phase 1; the creation of the Soaper Stars. Phases 2 and 3; the hospital trials (research questions I and II). Phases 4 and 5; the school trials (research question III).

Taking a mixed methods approach, this study gathered quantitative and qualitative data, employing observations of paediatric patients, and questionnaires and interviews with HCW across five hospital sites. Pre-and post-intervention worksheets, observations and knowledge checks were conducted with children in four schools and feedback was gathered from IP nurses working in schools (Figure 2).

### 3.1 Creation of the Soaper Stars

The creation of ‘superhero’ characters associated with a hand hygiene product came out of a collaborative process between the Germ’s Journey team and PAL International. Further development and refinements were made with the support of a focus group made up of De Montfort University post graduate students, undertaking a Masters in Education Practice degree. A range of educational resources underpinned by learning theories were designed to accompany the products.

Leading children’s hospitals were recruited to assist in planning a trial to evaluate the use of the products and educational resources in promoting good hand hygiene on paediatric wards.

### 3.2 Recruitment of hospitals and schools

NHS hospital trusts were initially selected based on their links with the study team and PAL International, to gauge their interest in trialling the Soaper Star HH boxes and to be involved in the development of the trial design. Once the trial had received ethical approval, it was published on the ISRCTN Registry as well as being promoted at infection prevention and control (IPC) conferences to recruit additional NHS hospitals. The Nuffield hospital group (private hospitals) expressed interest in leading their own trial of the Soaper Star boxes. The trial was carried out in a total of four NHS hospitals and one Nuffield hospital across the country.

Primary schools from around the East Midlands that had previously hosted Germ’s Journey workshops were invited to take part in the trial. The Nuffield group also approached primary schools with whom they had established links.

### 3.3 Recruitment of participants

#### 3.3.1 Phase 2—NHS hospitals

The researchers worked closely with the ward managers to recruit suitable participants based on the inclusion and exclusion criteria of the trial; that is, patients over 3 years old, who did not have an infectious illness, and preferably those who would be eating (patients who were not ‘nil-by-mouth’). It was also important that the researchers considered other criteria, such as patients who had mental health issues and eating disorders, in which being observed during mealtimes would not be appropriate (Table 1).

**Table 1:** NHS patient inclusion and exclusion criteria.

Patient inclusion criteria	Patient exclusion criteria
Patients aged three-years-old and over Patients who had a parent/carer to consent for them Patients who were well enough to take part Patients who would be eating	Patients under three-years-old Patient over 15 years-old Patients who did not have a parent/carer to consent Patients who required isolation. Patients who were nil-by-mouth Patients with eating disorders or mental health issues in which nursing staff did not deem it appropriate to observe.

After participant selection, the ward manager then spoke to the parents and carers of the patients regarding the trial and established if they would like to be involved, they then introduced the research team to the parents/carers to answer any questions about the trial. The parents/carers were given oral and written participant information about the project and were asked to complete a consent form. Where possible, the paediatric patient was asked for their consent as well. Participants were recruited throughout the trial.

Care staff on the wards were asked to take part in interviews and questionnaires about their opinions of the Soaper Star boxes (Tables 2, 3).

**Table 2:** Overview of NHS hospital sample.

Hospital	Location	Paediatric participants (aged 5–10)	Health care staff	Population demographics in hospital’s immediate vicinity
1 (May ‘22)	Urban North England	11	3	6% from Black/Caribbean/mixed/other backgrounds; 27% from Asian background; 29% from non-British ‘white’ background; 38% from British white background
2 (July ‘22)	Rural South-West England	8	2	4.27% non-white; 8.6% non-British white; 87.13% white British
3 (Sept ‘22)	Urban East Midlands	2	8	7.8% from Black/Caribbean/ African background; 43.4% from Asian background; 7.9% from other/mixed background; 40.9% from White background
4 (Feb ‘23)	Urban West Midlands	12	4	8.9% Black/Caribbean/African background; 18.5% Asian background; 7.1% mixed/other background; 65.5% White background

Total number recruited participants (*n* = 57). Total number of participants who took part in the study (*n* = 39). Population demographics: This is representative of the populations within the immediate vicinity of the hospital site and may not be an accurate representation of the population served by each hospital. Not all those who identify as white are British or speak English as a first language.

**Table 3:** School sample of participants.

School	Number of participants (Aged 7–9)		Location	IMD Quintile	SEN support	Not English first language	Free school meals
1	Year 3	*n =* 80	Rural East Midlands	5	6.7%	3.2%	3.7%
2	Year 3	*n =* 28	Rural East Midlands	5	5.6%	9.3%	7.9%
3	Year 3	*n =* 55	Urban East Midlands	1	13.4%	30%	44%

This table gives an overall picture of the school catchment, and the pupils who participated in the workshops.

#### 3.3.2 Phase 3—Leeds Nuffield hospital

The Leeds Nuffield hospital recruited patients for the trial, who either attended an outpatients’ clinic or were in-patients on a paediatric ward.

#### 3.3.3 Phase 4 and 5—school participants

Participants were selected based on their age. The workshops were conducted with children in Year 3 (aged 7–8-years) and Year 4 (aged 8–9-years) (Table 3).

### 3.4 Trial design

#### 3.4.1 Phase 2—NHS hospital trial

Over 3 days, the researchers observed the hand hygiene practices of the paediatric patients during their midday and evening mealtimes. Patients and/or their parents/carers would collect their food from a trolley wheeled into the ward, or a Care Assistant would take the meal to the patients’ beds. The standard practice in all four hospitals was to place a sachet containing a hand wipe on each tray (Table 4).

**Table 4:** NHS Hospital trial; Three day intervention study design.

Day	Intervention
1	Non-intervention No products or educational resources. Researchers observed typical hand-hygiene practices around mealtimes, monitoring the use of hospital-supplied hand wipes. With the support of housekeepers, the number of meals supplied to patients and therefore the number of wipes distributed were recorded. When the trays were collected by the ward housekeepers after the meals, the number of used wipes were recorded again, showing the number of patients who used their wipe or not.
2	Partial intervention Participants were given Soaper Star products (liquid soap, hand sanitiser, hand wipes) only, no educational resources. Researchers did not engage with the participants, they were observed discreetly to measure the use of the products at mealtimes.
3	Full intervention Full soaper star box including hand products and educational resources (puzzle sheet, poster, colouring pencils and sticker). Participants and their parents/carers were given the boxes by a Germ’s Journey team member and discussed the importance of hand-hygiene. Hand-hygiene practice and use of the products were recorded, `and participants were asked for any feedback on the boxes.

#### 3.4.2 Phase 3—Leeds Nuffield hospital trial

The Leeds Nuffield hospital conducted their own trial of the Soaper Star boxes in a day-clinic setting and on a ward. Following a similar structure of the three-day intervention, participants were placed into one of three groups over the course of 1 day. Ten of the participants were only given the products (liquid soap, hand sanitiser and wipes; group 1), 13 were given the whole box but there was no intervention or discussion about HH (group 2), and six patients received the whole box and were engaged in conversation about hand hygiene (group 3) Table 5.

**Table 5:** Leeds Nuffield hospital trial three-group intervention study design.

Group	Intervention
1	Minimal intervention Participants were only given the hygiene products (liquid soap, hand sanitiser, hand wipes).
2	Partial intervention Participants were given the complete boxes of hygiene products and educational resources but were not engaged in any intervention or discussion.
3	Full intervention Participants were given the complete boxes and were engaged in discussion about the importance of hand-hygiene, and best handwashing practice (using the healthcare worker questionnaire to guide their discussion).

#### 3.4.3 Phase 4—East Midlands school trial

**Figure 3:**
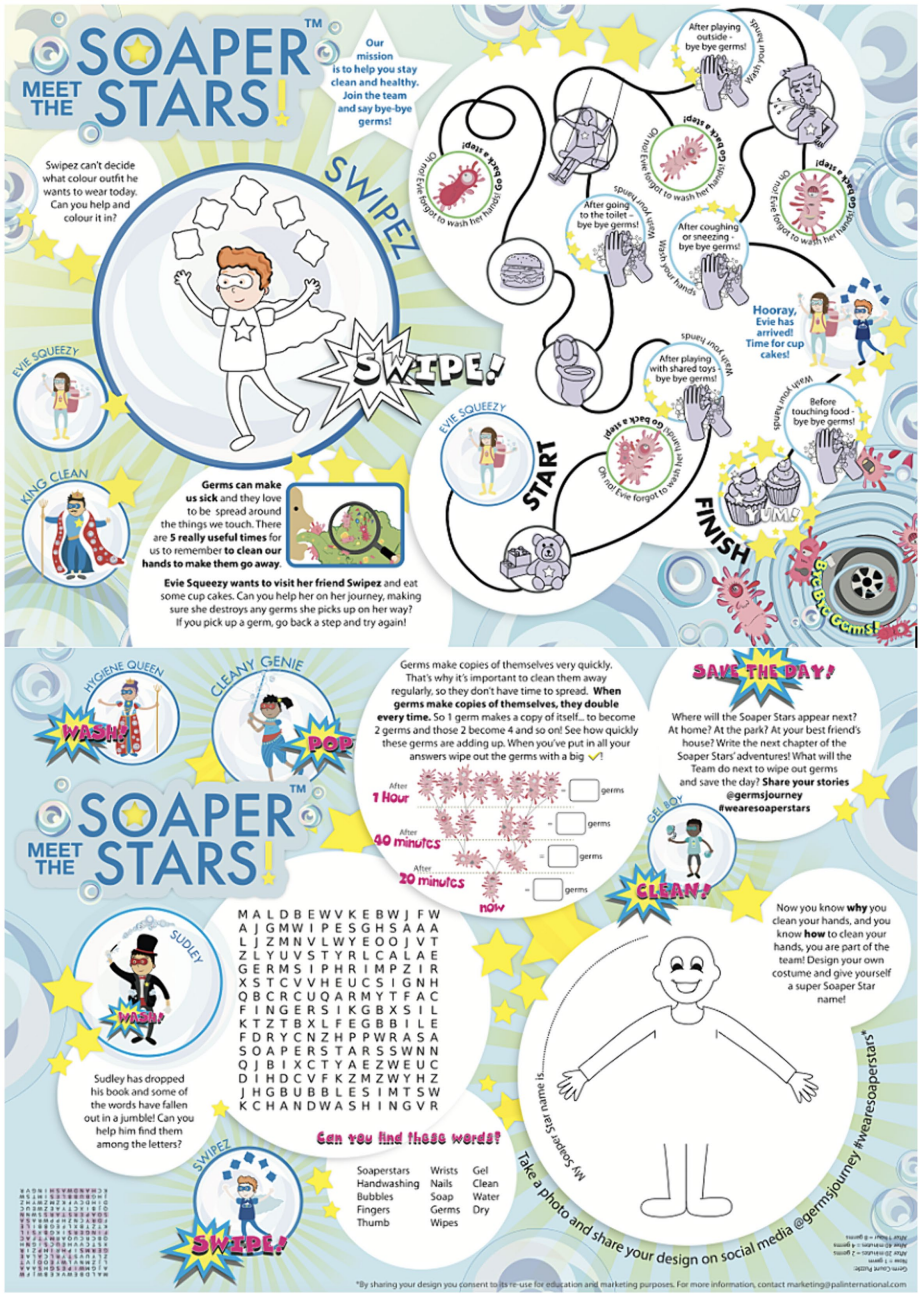
The Soaper Stars activity sheet.

**Figure 4:**
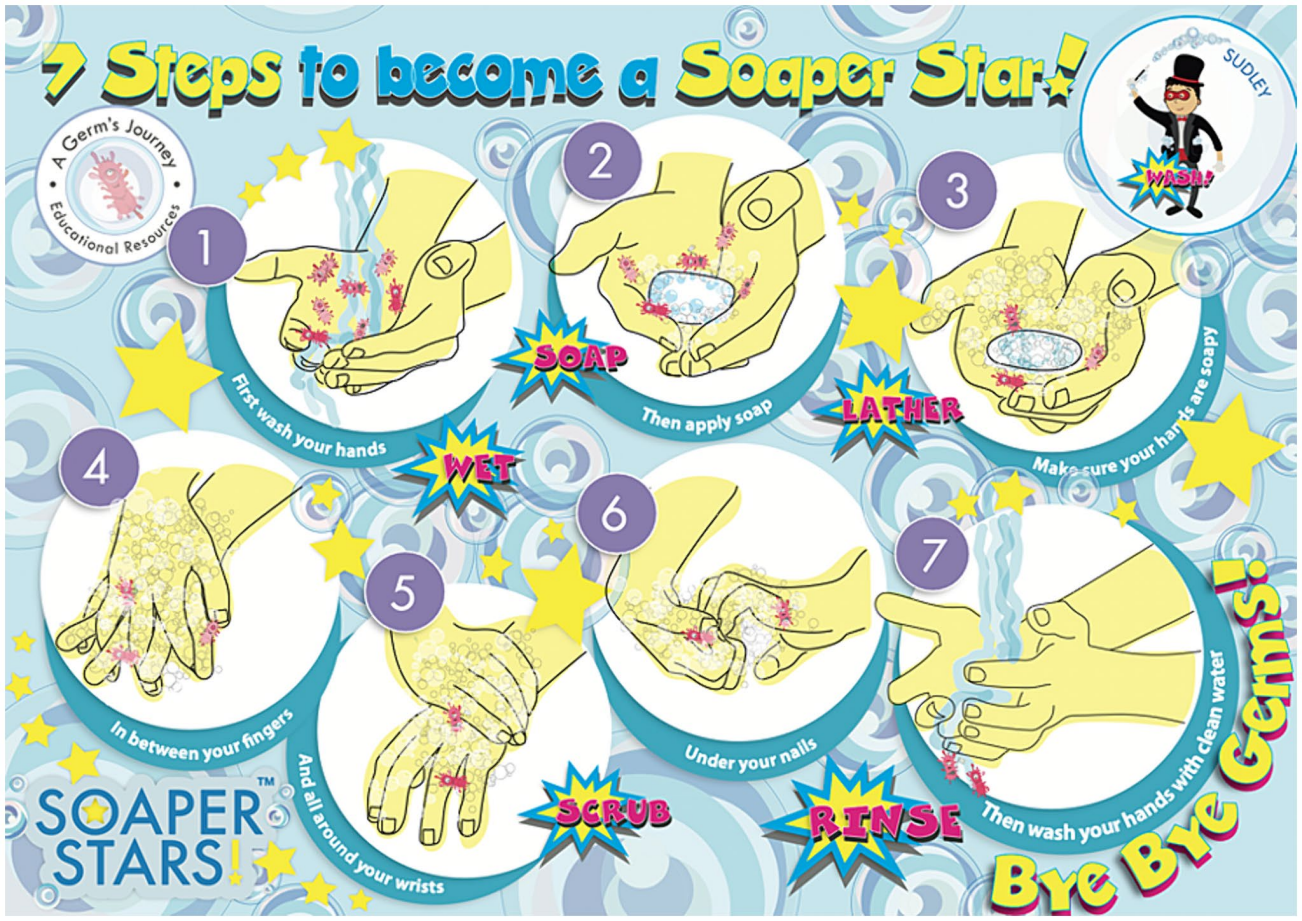
The Soaper Stars handwashing poster.

Educational workshops, underpinned by pedagogic theories of learning, and designed to meet the National Curriculum links for Key Stage 2, were delivered to three schools. The workshop took a whole-class approach and lasted approximately 1 h, beginning first by introducing the Soaper Star characters (Figure 1) and giving the pupils time to look at the boxes and their contents (Figures 3, 4). Next, the researchers spoke to the class about why handwashing is important, and explained the different types of germs (bacteria, viruses and fungi) and the concept of ‘good’ and ‘bad’ germs, including an overview of bacterial replication through binary fission using the worksheet (Figure 3) and videos. Following this, pupils were taught when to wash their hands, taking part in a ‘maze’ activity on the worksheet that showed occasions when hand hygiene is important (Figure 3). The pupils were also asked to think of other occasions when handwashing is necessary that were not included in the activity. Lastly, how to effectively wash hands was discussed, using the handwashing poster as a guide and reminder of the 6-step hand washing method recommended by the WHO (Figure 4).

#### 3.4.4 Phase 5—Leeds Nuffield hospital school workshops

The Leeds Nuffield hospital that took part in the hospital trial also lead IP workshops in schools, as part of their commitment to community outreach. IP nurses lead workshops in local schools with year 1 and year 4 pupils using eBug® activities, supported by the Soaper Star boxes, using the products and the educational resources.

### 3.5 Data collection tools

#### 3.5.1 Phase 1—NHS hospitals

##### 3.5.1.1 Observations

Non-invasive observations were carried out by the research team, to observe paediatric patients’ HH behaviours around their midday and evening mealtimes. The research team observed firstly whether the patients took part in any HH practices when no intervention was in place, then again when just the Soaper Star products were given to the patients, and then finally again when they had been given the full Soaper Stars box, with the products and educational resources. The observations also looked at how the patients interacted with the educational resources, and gathered feedback from parents about it. Researchers observed from a distance, so that they could see the patients but so they were not obvious in their observations. The researchers also did not engage in conversation with the children.

##### 3.5.1.2 Feedback from families and healthcare staff

Parents/Carers of the participants and hospital HCW were invited to give their feedback on the Soaper Star boxes, whether they thought the boxes were engaging, if the boxes would increase the children’s understanding and encourage the children to improve their HH practices.

##### 3.5.1.3 Questionnaires and interviews

Boxes were given out on the wards for approximately a month following the initial trial. Ward staff including nurses and housekeepers, were invited to complete questionnaires or to be interviewed for their observations and experiences of patients’ HH practices on the ward, and the potential effect of the Soaper Stars boxes on the HH of the patients. The interviews took a semi-structured approach using the questionnaire questions as a guide.

#### 3.5.2 Phase 3—Leeds Nuffield hospital

Feedback was collected from participants and their families by nurses using the questionnaire as guidance and prompts.

#### 3.5.3 Phase 4—East Midlands school trial

Pupils were given a brief worksheet containing open-ended and multiple-choice questions, to measure their understanding of the ‘why, when and how of hand hygiene’ immediately before and after the workshop, to see if the workshop had any impact on their knowledge post-intervention. The worksheet was repeated a month later, to assess if any of the knowledge had been retained.

#### 3.5.4 Phase 5—Leeds Nuffield school workshops

##### 3.5.4.1 Observations and knowledge checks

For the Leeds Nuffield Hospital school workshops, knowledge checks were made at various points during the workshops through oral question and answer sessions to measure the children’s understanding.

##### 3.5.4.2 Interviews and questionnaires

Qualitative data was also collected from the nurses leading each workshop session, who gave their opinions and feedback on the boxes, using the healthcare worker questionnaire as a guide (Table 6).

**Table 6:** Data collection tools overview.

	Data collection tools
Phase 2—NHS Hospital Trial	Observations Feedback from Families Questionnaires and Interviews
Phase 3—Leeds Nuffield Hospital Trial	Observations Feedback from Families and Healthcare Staff Feedback from Nurses
Phase 4—School Trial	Pre and post-intervention worksheets
Phase 5—Leeds Nuffield Hospital School Workshops	Observations and knowledge checks Nurses’ Feedback

### 3.6 Data analysis

#### 3.6.1 Quantitative data

##### 3.6.1.1 Phase 2—NHS hospital trial

The number of participants who used the Soaper Star products in each hospital was compared with the number who had used the hand wipes supplied by the hospital. Statistical analysis was undertaken using a Pearson’s Chi-Squared test using IBM SPSS Statistics for Windows, Version 28.0 (Armonk, NY). Significance was set at *p* ≤ 0.05.

##### 3.6.1.2 Phase 3—East Midlands school trial

For the multiple-choice questions in the worksheet, the frequency with which each option was selected was counted, differences in the frequency from immediately before and after the workshop, and in the four-week follow-up were analysed. Statistical significance was tested with Pearson’s Chi-Squared test, using IBM SPSS Statistics for Windows, Version 28.0 (Armonk, NY). Significance was set at *p* ≤ 0.05.

#### 3.6.2 Qualitative data

Responses to open-ended questions gathered during the questionnaires, interviews or from field notes during observations were examined for common themes and patterns following a thematic analysis framework (35). Responses to open-ended questions in the children’s pre and post worksheets from the school trial were grouped thematically, the number of responses in each group was compared.

## 4 Results

### 4.1 Phase 1—creation of the Soaper Stars

The Germ’s Journey team worked alongside PAL International, due to PAL’s interest in becoming more education-focused, using the United Nation’s Sustainable Developmental Goals (SDGs) as a basis for the direction of the education promotions. Germ’s Journey has contributed to SDG 3: Good Health and Wellbeing, SDG4: Quality Education, SDG16: Peace, Justice and Strong Institutions and SDG 17: Partnerships for the Goals (36).

Initial discussions between Germ’s Journey and PAL began in December 2018 to discuss how both teams could work together to promote HH and infection control, in both the United Kingdom and internationally, through a co-creation process. The idea for child-sized versions of PAL HH products, with each one linked to a ‘hygiene superhero’ arose. The diverse group of superhero characters, known collectively as ‘The Soaper Stars’ would be used in accompanying educational resources to engage children in different aspects of HH and infection control. The characters were designed to appeal to all children, with each character representing a range of ethnicities.

As part of the co-creation process, a focus group was undertaken with students studying a Masters in Education Practice at De Montfort University. These students included university academics, teachers, and postgraduate education students on placement in primary schools. The focus group allowed the researchers to gain an insight into what types of characters, illustrations, resources and products would be best suited to aid children’s learning on HH. Generally, the response to the characters was positive, with some suggestions on how the characters could be made more child-friendly. Originally, it was intended that the leader of the Soaper Stars would be female (‘the Hygiene Queen’). It was suggested that some countries be more familiar with a male authority figure, so a king (‘King Clean’) was added, with King Clean and Hygiene Queen being joint leaders. The original character designs shown to the group were quite ‘cartoonish’ in style, however the group recommended a more realistic appearance would be more appropriate.

**Figure 5:**
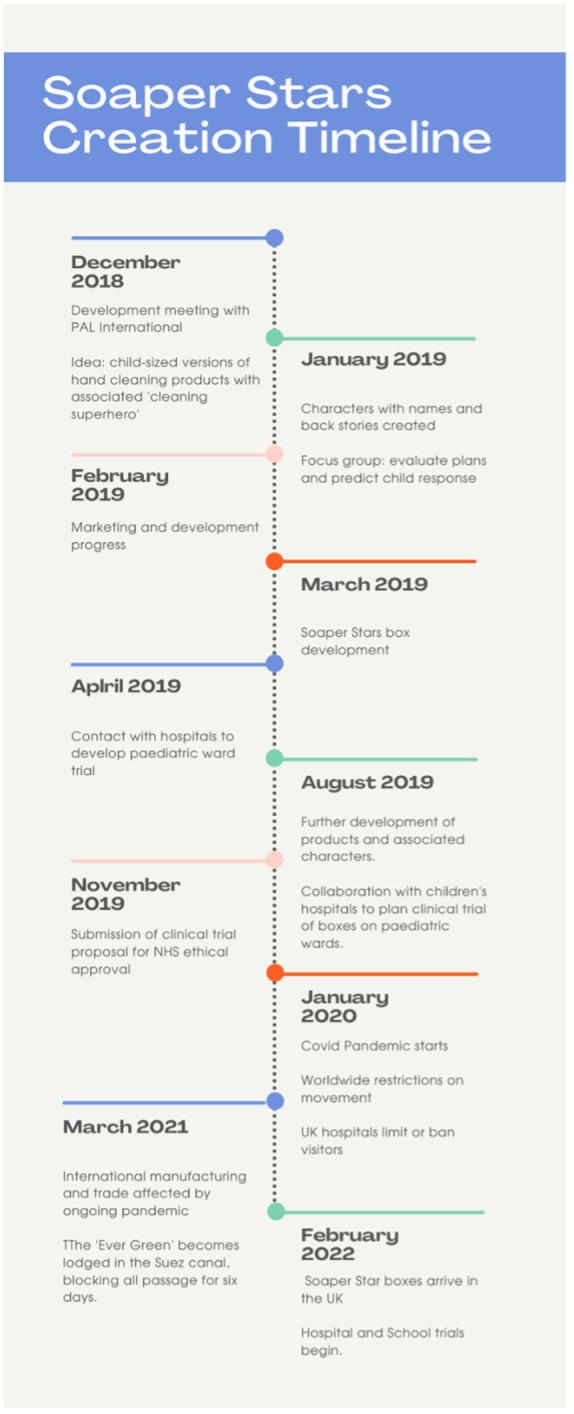
Creation of the Soaper Stars timeline.

The hospital trial was created with two IP nurses from prominent children’s hospitals. and submitted for NHS ethical approval in November 2019, with the intention of starting the research in February 2020. The outbreak of the COVID pandemic in January 2020 led to the closure of hospitals to visitors, and eventually to the restriction of movement across the country. After some logistical issues in manufacturing and distributing the Soaper Star boxes following the Suez Canal blockage (37), and once Covid restrictions had eased, permission had been granted by both the research team’s University and the NHS study sites for research to resume (Figure 5). The first hospital trials started in May 2022. School-based workshops using the products were also delivered during 2022.

### 4.2 Phase 2—NHS hospital trial

#### 4.2.1 Observations

Observations were carried out on the paediatric wards to measure the patients’ engagement in hand-hygiene practices and the specifically developed Soaper Star HH boxes. Participants were recruited each day, with the support of the ward manager or the lead research nurse. Not all participants that were recruited on the first day were able to participate in the subsequent days due to the patients either being discharged, or if their condition had deteriorated.

##### 4.2.1.1 Day 1: No intervention

For the first day of the intervention, the researchers observed general practice on the wards. All hospitals provided patients with wet wipes in plain sachets on food trays with their meals. For this trial, the housekeepers on each ward kept a record of how many sachets of wet wipes were distributed and how many had been used when the trays were collected after the meal (Table 7). These numbers include all ward patients who had food, not just those who were participating in the Soaper Stars trial. The housekeeper at Hospital 2 stated that it was quite common for wipes to be returned unused unless a specific comment was made when the tray was handed over to the patient, and even then, most patients still did not use the wipes given. This is reflected in the data collected in this study, in which hospitals 1 and 2 had 12.5% (1 of 8) and 25% (2 of 8) wipe usage, respectively, while none of the 11 wipes at hospital 3 were used. In contrast, hospital 4 had 40% (10 of 25) wipe usage (Table 7).

**Table 7:** Comparison of the number of hospital wipes distributed and used, with the number of Soaper Star products, and complete Soaper Star boxes.

	Day 1: Hospital wipes			Day 2: Product only				Day 3: Complete box			
Hospital	Handed out	Used	% used	Handed out	Used	% used	% difference from day 1	Handed out	Used	% used	% difference from day 1
1	8	1	12.5	7	5	71	58.5	7	3	43	30.5
2	8	2	25	4	2	50	25	8	4	50	25
3	11	0	0	2	2	100	100	1	1	100	100
4	25	10	40	6	3	50	10	8	4	50	10
All hospitals combined	52	13	25	19	12	63	38*	24	12	50	25*

*Statistically significant difference, *p* ≤ 0.05.

##### 4.2.1.2 Day 2: Part-intervention (hand-hygiene products only)

On day 2, participating patients received the hand hygiene products only from the Soaper Star boxes; that is, liquid soap, a packet of wet wipes, and hand sanitiser. The products were placed on bedside tables and there was no engagement with the patients or their families in conversation about hand hygiene, to see the effectiveness of the products alone.

At least half the participants in each hospital used the Soaper Star products that were distributed on Day 2, amounting to 63% of participants across the four hospitals, compared to just 25% using the hospital-supplied wipes. Demonstrating an increase in the use of hand hygiene products around meal times when the Soaper Star HH products were introduced (*p* ≤ 0.05; Table 7).

##### 4.2.1.3 Day 3: Full intervention (complete boxes)

Participating patients were given the complete Soaper Stars HH boxes which included the products and the educational resources (posters, activity sheet, sticker and colouring pencils). Once the Soaper Star boxes were distributed, families were invited to give feedback on the boxes. With regards to usage, there was a small decrease (13%) on the third day compared to day 2, with 50% (12 participants) of the participants across all four hospitals using the products in the complete boxes. Again, the data showed a significant (*p* ≤ 0.05) relationship between the product and its use (Table 7).

#### 4.2.2 Feedback from parents/carers

The parents/carers of the participants expressed positive opinions on the boxes, with one parent commenting that *“These are a really good idea, much more child friendly”* (parent of patient 1, Hospital 1). Similarly, another parent noted that, *“The box is very attractive; the wet wipes are very helpful to encourage my child to clean their hands.”* (Parent of patient aged 10, Hospital 4). Another comment included: *“The packs are very useful for parents as well as the children, especially the wipes and sanitiser. We sometimes help with changing dressings, and it is useful to have the products to hand”* (Parent of patient aged 6, Hospital 4).

#### 4.2.3 Interviews and questionnaires

A total of nine staff consisting of five nurses and four housekeepers (one from each hospital) took part in interviews or completed the questionnaire. Findings from the interviews/questionnaire demonstrated that the Soaper Stars HH boxes were thought to be designed well, with the staff responding positively.

Findings from the interviews/questionnaires have been organised into three themes.

##### 4.2.3.1 Previous hand-hygiene interventions

When asked about previous hand-hygiene interventions within the hospitals aimed at children, only 1 member of staff had previously taken part in one. They noted that the campaign had taken the form of posters and images on soap dispensers, but they did not think it had been particularly effective. They further noted that the boxes were seen to be more likely to engage the interest of the children.

##### 4.2.3.2 The use of the boxes on the wards

Hospital 4 distributed the boxes in the day clinic adjacent to the paediatric ward; the nurse who distributed them reported that the boxes were positively received and engaged a lot of interest, with the stickers and activity sheets being particularly popular. One nurse noted that the wipes and the hand sanitiser tended to be used more than the soap, commenting further that “*a lot [of children] are poorly and in bed, hooked to IV (Intravenous) drip* etc. *So, wipes and hand gel are more efficient. If used outside of hospital, soap would be used more.,* e.g.*, if put into lunch boxes*” (IP nurse, Hospital 4). The IP nurse in Hospital 4 added that that handwash basins were hard to reach for the children, further reducing the opportunities for handwashing.

When asked whether they thought the frequency of handwashing has increased following the distribution of the Soaper Star boxes, four of the healthcare staff (44%) stated yes, two (22%) felt there was no change, the remaining third (3–34%) were unsure whether the frequency of the hand cleaning had been affected.

##### 4.2.3.3 The role of parents in hand-hygiene education

The staff highlighted the importance of parental involvement when teaching children about hand-hygiene, with one IP Nurse commenting: *“Parents are the way, if you can get them on board. They can be a barrier or an enabler, so they are very important in promoting hand hygiene.”* (IP nurse, Hospital 1). Another noted that the boxes were *“a prompt for both nurses and parents to encourage hand cleaning” (nurse, Hospital 4)*.

### 4.3 Phase 3—Leeds Nuffield hospital trial

#### 4.3.1 Observations

##### 4.3.1.1 Engagement with Soaper Stars HH boxes

Boxes were handed out to patients in a day-clinic setting and on a paediatric ward. When measuring the participants’ engagement with the Soaper Star boxes, most (22 of 29, 76%) of the participants were interested with the contents of the Soaper Star boxes. Participants wore the stickers, looked at the posters and used the activity sheet and/or the hand products.

The results found that 24% (7) of participants did not engage. Five participants were in group 1 (who received the products only). The nurses’ observations were that the products had been pushed to one side by the participants or the adult with them, or the child was using an electronic device and did not acknowledge the products. One family stated that the child was too anxious, and they were focused on keeping them calm. The other two participants were each in the remaining groups, Group 2—partial Intervention (where children received the full boxes but were not involved in any discussion) and Group 3—full intervention (where Participants were given the complete boxes and were engaged in discussion about the importance of hand-hygiene). However, these two children were aged 13 and 14, and therefore, were older than the target age group of the resources. Overall, the data showed that 69% (20 of 29) of the participants used the products. Table 8 shows the participants’ engagement, the products used and their favourite characters.

**Table 8:** Leeds Nuffield hospital participant engagement in Soaper Stars *HH* boxes.

	Group	% engagement*	Comments on engagement	Products used	Favourite characters
1	Products only *(n = 10)*	50	5 made use of products, 5 did not show interest in products	Gel: 5 Soap: 2 Wipes: 3	Evie Squeezy = 2 Gel-Boy = 3 n/a = 5
2	Whole box, no intervention *(n = 13)*	84.62	11 used products and resources, 1 used activity sheet only, 1 showed no interest—aged 14	Gel: 11 Soap: 5 Wipes: 2	Evie Squeezy = 5 Gel-Boy = 4 n/a = 5
3	Whole box and intervention *(n = 6)*	66.67	4 used products and resources, 1 used activity sheet and stickers only, 1 showed no interest—aged 13	Gel: 3 Soap: 4 Wipes: 3	Evie Squeezy = 3 Gel-Boy = 1 Swipez = 1 King Clean = 1 n/a = 1

*Engagement indicates use of the hand hygiene products, and/or the educational resources. Some patients used more than one product. The table shows the groups at the Leeds Nuffield hospital and the level of engagement with the Soaper Stars.

##### 4.3.1.2 Soaper Stars HH boxes effect on paediatric patients’ handwashing and understanding

The nurses at the Leeds Nuffield Hospital noted a positive effect on children’s HH practices, reporting an increase in the frequency of handwashing. Children tended to use the soap and sanitiser more than the wipes. Parents commented that the children would take more time to wash their hands, paying closer attention to handwashing actions. Some of the children from groups 2 and 3 who received the whole box, were observed to be following the handwashing stages depicted in the handwashing poster provided in the box.

##### 4.3.1.3 Understanding of germ transfer and handwashing

Following their engagement with the products and resources, the children were able to explain about the importance of hand-hygiene. Children noted that: *“I need to wash my hands before I eat”* (group 3 participant); *“wash, wash, scrub my germs away”* (group 3 participant); *“I need to wash my hands when I’ve been for a wee”* (group 2 participant); *“need to scrub, scrub, scrub my germs away”* (group 1 participant).

##### 4.3.1.4 Feedback from parents

Nurses at the Leeds Nuffield Hospital spoke to 29 of the children’s parents/carers and asked them for some feedback about the boxes, 18 (62%) of which gave positive feedback, stating that they thought the boxes the boxes were a good distraction for their children who were waiting for medical treatment, with one noting that it was “*good to have something to take his mind off the waiting.”* Other parents/carers commented positively on the “*handbag-friendly”* size of the boxes, with others also describing the boxes as child-friendly, noting that the products had a good smell and attractive presentation that would appeal to the children and make them excited to wash their hands. Of the 29 parents, 11 (38%) did not comment.

#### 4.3.2 Interviews and questionnaires

The nurses also provided their feedback, commenting that the boxes provided them with an opportunity to highlight the importance hand-hygiene with the families, further commenting that the products were easy to use, and the resources were helpful for the nurses, with one nurse explaining: *“[it is] a great resource, the [handwashing] poster helps to visualise the steps for children, and they can do it together with parents.”*

### 4.4 Phase 4—East Midlands school trial

Three schools that had previously hosted Germ’s Journey workshops agreed to participate in the trial. Table 3 shows key characteristics of the schools. The index of multiple deprivation (IMD) is calculated from a range of social and economic factors based on the postcode of the school. The most deprived areas are scored at 1, with the least deprived areas scoring 5. School catchment areas may cover a range of IMD scores. School 1 is located on the outskirts of a small town, school 2 is in a rural village setting, and school 3 is on the border of a city.

A total of 223 pupils took part in the workshops, with 60 in year 4 (aged 8–9) and 163 in year 3 (aged 7–8). The responses of the year 4 pupils on the pre-workshop worksheet showed very good knowledge and understanding of HH therefore only the responses of the year 3 pupils are reported here.

#### 4.4.1 Pre and post workshop questionnaires

The results from the worksheets that children were asked to complete directly before and after the workshops are presented below. The worksheets included questions about the why, when and how of handwashing.

##### 4.4.1.1 Why do we need to wash our hands?

Immediately before the workshop, children were asked to write their reasons for washing their hands, with 94% (154) of the pupils’ answers referring to washing germs away, or to stay clean and healthy, with some children specifically mentioning ‘covid-19’ or ‘the pandemic’. 2.2% (5) pupils referred to using soap and water or ‘smelling nice’ and 1.8% (4) pupils did not answer. The same question was asked, but this time provided multiple choice answers, of (a) to make your hands grow, (b) to wash away germs and dirt, (c) to make our skin soft. Two pupils (1%) did not answer, and 161 pupils (99%) selected the correct answer (answer b: to wash away germs and dirt). Responses immediately after the workshop, and in the four-week follow-up were very similar.

##### 4.4.1.2 When should we wash our hands?

Pupils were given eight options for when it is most important to wash their hands and were told that they could select as many as they liked. The responses to each question, before, after and a month following the workshop are presented in Table 9.

**Table 9:** When should we wash our hands? Combined responses from year 3 pupils at all three schools.

	Pre workshop		Post workshop			1 month follow up			
Options	N	%	N	%	Difference	N	%	Difference with pre w/s	Difference with post w/s
When we get out of bed.	5	3.07	8	4.91	1.84	19	11.66	8.59	6.75
Before eating or handling food.	148	90.8	150	92.02	1.23	156	95.71	4.91	3.68
After reading a book.	8	4.91	27	16.56	11.66	22	13.50	8.59	−3.07
After playing with shared toys.	109	66.87	135	82.82	15.95	146	89.57	22.70	6.75
When we come in from outside.	127	77.91	143	87.73	9.82	151	92.64	14.72	4.91
When we have been watching TV.	3	1.84	8	4.91	3.07	13	7.98	6.13	3.07
After going to the toilet.	150	92.02	152	93.25	1.23	152	93.25	1.23	0.00
After coughing or sneezing into our hands.	150	92.02	150	92.02	0.00	156	95.71	3.68	3.68

The frequency of responses is given as a percentage of the total.

##### 4.4.1.3 How should we wash our hands?

Pupils were asked ‘how should we wash our hands’ and given six options to select based on what they thought was the best method. Like before, they could choose more than one option. The number of children choosing the correct handwashing methods after the intervention increased significantly (*p* < 0.05) compared to the pre-intervention data.

The responses to each question, before, after and a month following the workshop are presented in Table 10.

**Table 10:** How should we wash our hands? The combined responses from year 3 pupils at all three schools.

	Pre workshop		Post workshop			1 month follow up			
Options	N	%	N	%	Difference	N	%	Difference with pre w/s	Difference with post w/s
With soap and clean water	158	96.93	149	91.41	−5.52	157	96.32	−0.61	4.91
With hand gel/sanitizer	97	59.51	114	69.94	10.43	128	78.53	19.02	8.59
With a dry tissue	7	4.29	8	4.91	0.61	6	3.68	−0.61	−1.23
With a wet-wipe	60	36.81	100	61.35	24.54	108	66.26	29.45	4.91
With a dirty towel	1	0.61	4	2.45	1.84	2	1.23	0.61	−1.23
With a puddle of water	4	2.45	6	3.68	1.23	2	1.23	−1.23	−2.45

As part of the worksheet, children were also asked to draw circles on an illustration of the front and back of a hand. The circles were to indicate the most important areas to pay attention to when washing hands. There was no discernible pattern in the responses. Many children simply drew a large circle that encompassed the whole hand, others covered the drawing with small circles, and some made no attempt at all.

### 4.5 Phase 5—Leeds Nuffield school workshops

#### 4.5.1 Observation and knowledge check data

The nurses who lead the workshops noted that the pupils were all very excited to open the boxes and investigate the contents, with the stickers being particularly popular. The nurses stated that the handwashing poster was particularly helpful in teaching the stages of handwashing noting that: *“[the handwashing is] more thorough.”* (IP nurse 1 working with KS1 class) *“[the pupils] took time to complete all the steps.”* (IP nurse 2 working with KS1 class). It was also reported that the activity sheet helped children to understand the importance of hand hygiene. The pupils’ baseline knowledge regarding why and how to wash hands was generally good. However, the nurses noted that their understanding of when to wash hands was not so good, with one nurse commenting that that the pupils did not know that they should wash their hands when they were ill.

#### 4.5.2 Nurses’ feedback

Using the questions from the Healthcare workers’ questionnaires as a guide, the Leeds Nuffield IP nurses who led the school workshops gave feedback regarding the boxes’ usefulness for teaching children, stating that they found the boxes to be a great resource in reaching out to the community and supported them in helping the pupils to understand the importance of good hand-hygiene. Comments included: *“[the] children are washing their hands more often, reducing infection.”* (IP nurse 1); *“[the boxes support] reaching out to the wider community for classroom sessions.”* (IP nurse 2); *“[the boxes are] great resources, the gel was well received, the posters to identify steps.”* (IP nurse 3).

## 5 Discussion

### 5.1 Phase 1—creation of the Soaper Stars

The Soaper Stars were created as part of an ongoing project to educate children about the importance of HH (30), focusing on the ‘why, when and how’ of HH. Underpinned by theories of learning and scientific principles, the Soaper Stars educational resources and HH product boxes were developed, following Kolb’s theory of Experiential learning (38), Vygotsky’s theory of learning through play (39), Skinner’s behaviourist theory of positive reinforcement (40) and the COM-B model (41).

Experiential learning argues that ‘hands-on’ learning enables the child to learn abstract concepts (in this case, pathogen transmission) and translate that into ‘real-life’ learning, in that, the concepts are made relevant to the children, and feel more real (38). Likewise, Vygotsky’s advocacy for learning through play as a vehicle to enhance children’s cognitive development was considered when developing the puzzle activities in the box (39). Skinner’s theory of positive reinforcement notes that a desired behaviour is more likely to be achieved if this behaviour results in a ‘reward’, which subsequently leads to the repetition of such behaviour (40). In this instance, by children understanding why they should partake in HH practices, and the benefits of such, alongside being given an attractive and fun box, equipping them with the information and ‘tools’ (products) to do so, children are more likely to comply in HH practices. Similarly, the box of HH products alongside the learning resources encourages and supports behaviour change according to the COM-B model. This model notes that for behaviour change to occur, the individual requires the capability, opportunity and motivation to do so. The resources support the capability and motivation to improve hand hygiene, while the products support the opportunity for it to take place (41).

### 5.2 Phases 2 and 3—Soaper Stars hand-hygiene intervention in hospitals

HAIs are a global problem, affecting as many as 15% of acute care patients. The European Centre for Disease Prevention and Control (ECDC) estimates this could be 4.5 million cases every year across Europe alone (1). Many of the infections are caused by antimicrobial resistant bacteria, such as methicillin-resistant *Staphylococcus aureus* (MRSA), with Carbapenem-resistant and Extended spectrum β-lactamase producing *Enterobacteriaceae* being particularly associated with paediatric HAIs. Good hand and environment hygiene are essential in breaking the chain of transmission and reducing the rate of infection (42). It is estimated that these interventions could reduce the rate of infection by as much as 70%, and provide healthcare cost savings of US$1.65 for every US$1 invested (43).

IP interventions have focused on the HHC of HCWs, with multimodal approaches being more successful than single strategies (44). Patients and visitors are now being encouraged to participate in IP by improving their HH predominately through reminders, either verbal or written (9–11, 14).

Paediatric patients are often overlooked in hospital IPC intervention studies so there is a lack of data with which to compare the results obtained in this trial. The only similar intervention to this study is the Glo-yo gadget which uses Glo-gel based interventions with paediatric patients, their families and visitors to demonstrate effective handwashing (12). Though a fun approach for children, it required a 30 min training session to use. It is a gadget that teaches good technique but does not provide the means for maintaining good hygiene on a daily basis. As part of the Glo-yo trial, control groups were either shown a 30 min video or given a leaflet that took 2 min to explain, with the trial finding that the leaflet was as successful as the two interventions, in terms of having an impact on HH compliance during the trial (12).

The Soaper Star boxes, in contrast, contain familiar products that are readily recognisable and can be used without prior instruction, and with minimal English-language skills. Unlike the Glo-yo intervention, they provide the children with the means to keep their hands clean on a day-to-day basis, alongside the educational activities that help them to understand why HH is important. Therefore, it could be argued that the Soaper Stars are better suited to a hospital environment than the Glo-yo, as healthcare staff can hand the boxes to patients, without needing to spend periods of time explaining how to use the products. This is a particularly important factor in interventions being sustainable, as realistically healthcare professionals are extremely busy providing care for patients. The Soaper Star boxes were designed in such a way that they would not put extra time restraints on healthcare workers.

This trial found that the Soaper Star boxes have successfully stimulated a 25–38% increase in the frequency of hand-hygiene practices around mealtimes for the period of the trial. The feedback from all five hospitals stated that the boxes are engaging and useful in encouraging paediatric patients (and their families) to comply with hand hygiene recommendations. This shows that hand-hygiene products designed for children can result in greater interest in hand-hygiene and increase the likelihood of effective handwashing.

The trial in NHS hospitals was dependent on the availability of the suitable participants, which was a challenge. Participant numbers were unpredictable, all four hospitals had large numbers of patients, but many were not suitable for this trial. Patients were often too young (under 3 years of age), too ill to participate or their condition deteriorated so they were unable to continue with the trial. In the Leeds Nuffield hospital, the IPC nurses lead the trial within their own wards, affording them a different level of access to the participants and their families than was available to the Germ’s Journey research team in the NHS hospitals. There is little published evidence that describes the difficulties in recruiting patient participants to HH trials, both for NHS and private hospitals. As this trial focused on patients, while other studies have focused on visitors, a direct comparison between the number of participants is difficult.

Housekeepers were key during this trial. The design of the paediatric wards, with long communal wards, private rooms and small bays made observing in a discreet and non-intrusive manner more challenging. However, the ward housekeepers, being more familiar with the wards, were able to interact with the patients and their families with ease and were best placed to record the number of used hospital-issued wipes that were delivered with meals. The housekeepers play a crucial part on the wards, being responsible for maintaining the cleanliness, but also serve and clear away meals, and can be called upon to talk with and reassure patients (45). Housekeepers prove invaluable in supporting the HH of patients, yet nevertheless, alongside cleaning staff, seem to be overlooked with regards to cleaning and IP interventions or strategies (46, 47).

Parents and guardians, are also a key influence in children’s HH compliance, with one IP Nurse commenting: “[parents/guardians] can be a barrier or an enabler.” This was reinforced by the qualitative data from Leeds Nuffield hospital, where parents/guardians were observed to be moving products away from their children, preventing the children from using them. This is similar to studies in other areas which show that parental involvement can positively impact children’s education (48).

The Soaper Star HH products attracted more attention from the participants than the hospital issued wipes, as can be seen in Table 7, in which there was a 63% usage of Soaper Star products in comparison to a 25% usage of hospital wipes. These numbers only record use by the paediatric patient, there were three occasions where parents/guardians were observed using the products as well. The boxes were identified as being helpful for the families who were involved with the medical care of their children, such as changing dressings or administering medication. Using wash basins or finding hand sanitizer dispensers could mean leaving the child unaccompanied, whereas having the box with products to-hand, enables the parent to maintain their own HH both before and after attending to the needs of their child (therefore, complying with the WHO recommended ‘five moments of hygiene’) (49). Studies show the importance of family care in healthcare settings in LMICs (16, 17) but there is limited published research on the importance of parental involvement within healthcare settings in the United Kingdom. That said, studies have shown the importance of parental involvement in children’s learning development (39, 48, 50–53).

Overall, the results of the hospital trials show that HH products specifically designed for children can lead to greater interest in HH and increase the likelihood of HHC among paediatric patients. The holistic approach of using a combination of educational resources that teach children the importance of HH, accompanied by the products that enable them to carry out HH practices was noted as being beneficial, according to parents and healthcare professionals within this hospital trial. This reinforces previous research that shows a holistic approach of supporting children’s knowledge of both why and how to wash hands, leads to improvements in their handwashing behaviour (30) and subsequently aids a reduction in illness (28).

### 5.3 Phases 4 and 5—Soaper Stars hand hygiene intervention in schools

Results from the school workshops found that children had a very high baseline knowledge that good HH is important, and were able to demonstrate good technique, perhaps not surprising, given that the children had lived through the Covid-19 pandemic. The older cohort of children (year 4, aged 8–9) had very high levels knowledge in the ‘pre-workshop’ worksheet (data not shown). However, the pre-workshop worksheet responses did demonstrate that there were gaps in the younger pupils’ understanding with regards to the ‘why, when and how’ to wash hands. Immediately after the workshops, there was a 15–22% increase in the knowledge around when to wash hands, and a 19–29% increase in knowledge around how to wash hands. These increases in knowledge were maintained for at least 4 weeks. The workshops, supported by the Soaper Star boxes of product and educational materials, provide useful tools for consolidating knowledge learnt through other sources, and filling gaps in the knowledge to give context for the prior learning (38–40).

Workshops started with ‘why do we wash hands’. Pre-workshop worksheet results show that the pupils knew that this was to stop the spread of germs, demonstrated by 96% of the pupils being able to give an answer linked to the removal of germs and stopping the spread of infection.

Although children had a high baseline with regards to ‘why’, pre-workshop worksheet data show that directly before the intervention, just over 1 in 5 (*n* = 36, 22.1%) did not know that they should wash hands after being outside. The National Curriculum states that schools are required to teach the importance of handwashing to all year groups as part of the statutory health education (54), and pupils had been observed washing their hands after being outside for their break, suggesting that although children did wash their hands after being outside, they did not necessarily understand why this is important. Data showed an increase in children selecting ‘when we come in from outside’ on their worksheets when asked when it is important to wash hands with 87.7% children selecting this after the workshop, compared to 77.9% beforehand. All options except for washing hands after reading a book saw an increase in selection in the follow-up quiz 1 month later, showing a retention in understanding the ‘when’ element of HH.

With regards to the ‘how’ of HH, the pupils, again, had a high baseline knowledge of this, with 96% of them selecting soap and water as the principal method for washing hands immediately prior to the workshop. Interestingly, less than 40% of the pupils selected wipes as a suitable method for cleaning hands before the workshop, but this increased to 66% in the 4 week follow-up.

It is not surprising, given that the children had lived through the Covid-19 pandemic, that most children had a good level of understanding of hand-hygiene. For these pupils, the Soaper Star workshops were more about reinforcing this knowledge and filling in the gaps, as opposed to introducing entirely new concepts. Reinforcing children’s learning is important to ensure that the understanding is embedded, and the desired behaviour is repeated (40). In addition, the COM-B model that was considered when developing the intervention encourages behaviour change (41).

Previous research highlights the benefits of school-based interventions (4, 23, 27, 30), and the influence that peer behaviour has on pupils (in that, children are more likely to wash their hands if their peers are doing so) (33, 34). By developing a class-based intervention, children learn with and from one another, and are more likely to complete HH practices if their peers are also doing so. Using the Soaper Star boxes to engage whole classes in the topic encourages pupils to model the good behaviour to each other.

The Leeds Nuffield school workshops also found positive results, with IPC nurses reporting that the workshops were popular with the children who were keen to investigate the boxes and share the contents. The nurses reported that their workshops helped the pupils to improve their handwashing technique, and their knowledge of germs, with the nurses finding the boxes helpful in engaging the pupils’ interest in the topic.

## 6 Conclusion

This research demonstrates that the Soaper Star boxes are an effective and attractive tool to engage children in HH. Involving science and education specialists in the co-creation process ensured the inclusion of scientifically accurate and age-appropriate information to support children in learning about the ‘why, when and how’ of hand hygiene. These boxes are the first of their kind in providing education resources alongside HH products in a form that is suitable for the healthcare environment, allowing paediatric patients to maintain their hand hygiene when receiving medical care. Families involved in the care of the children also appreciated the convenience of the boxes.

Using the boxes to support school based workshops reinforce children’s learning and filled knowledge gaps that were sustained for at least 4 weeks following the intervention. Overall, the results of this trial show that Soaper Stars combined education resources and products lead to significantly improved HH compliance in children, which is fundamental in controlling infectious diseases both in and outside of the healthcare arena.

## Data Availability

The datasets presented in this article are not readily available because the subjects of the study are under 18 years of age. Requests to access the datasets should be directed to p2628909@my365.dmu.ac.uk.
